# Multi-omics analysis to identify the dynamic changes of immune cells and marker genes in renal fibrosis

**DOI:** 10.3389/fgene.2026.1767093

**Published:** 2026-04-02

**Authors:** Wen Yi, Mingrong Cao, Xiaojun Tan, Guocheng Du, Jingdong Li

**Affiliations:** 1 Department of General Surgery, The First Affiliated Hospital, Jinan University, Guangzhou, China; 2 Department of General Surgery, Nanchong Central Hospital Affiliated to North Sichuan Medical College, Nanchong, China; 3 Department of Urinary surgery, Nanchong Central Hospital Affiliated to North Sichuan Medical College, Nanchong, China; 4 Hepatobiliary, Pancreatic and Intestinal Research Institute of North Sichuan Medical College, Nanchong, China

**Keywords:** ceRNA, exosome, macrophage, renal fibrosis, UUO

## Abstract

**Introduction:**

Renal fibrosis is a common pathological feature of chronic kidney disease and a major driver of progression to end-stage renal disease, but its molecular mechanisms remain incompletely understood.

**Methods:**

We integrated multi-omics datasets from GEO and published studies, including mRNA, protein, miRNA, and circRNA data from unilateral ureteral obstruction (UUO) models, TGF-β-induced in vitro fibrosis models, and human umbilical cord mesenchymal stem cell-derived exosomes (HucMSC-Exo). Differential expression analysis, functional enrichment, immune infiltration analysis, fuzzy c-means clustering, weighted gene co-expression network analysis, and ceRNA network construction were performed, with selected findings further validated experimentally.

**Results:**

We identified stable fibrosis-associated genes and proteins, with metabolic dysregulation emerging as a prominent feature of renal fibrosis. Time-series analysis revealed dynamic transcriptional changes during UUO progression. Comparative analysis showed that in vitro fibrosis models reproduced only part of the in vivo molecular landscape. Immune analyses consistently highlighted macrophages, especially M2-like macrophages, and also suggested a potential role for B cells. In addition, we identified immune-related hub genes and constructed fibrosis-associated ceRNA networks linked to macrophage regulation. Several miRNAs enriched in HucMSC-Exo, particularly miR-30a-5p, were predicted to counteract fibrosis, and exosome treatment alleviated renal injury, macrophage infiltration, and fibrotic marker expression.

**Conclusion:**

These findings provide a comprehensive view of the molecular and immune landscape of renal fibrosis, clarify key differences between *in vivo* and *in vitro* fibrosis models, and suggest potential therapeutic targets for antifibrotic intervention.

## 1 Introduction

According to the statistics, the global prevalence of chronic kidney diseases (CKDs) is 9.1%, as of 2017, the global prevalence of CKDs was 697 million (GBD Chronic Kidney Disease Collaboration, 2021). In the global list of total causes of death, CKD rose from 27th in 1990 to 18th in 2010, and CKD is the third leading cause of premature death, after AIDS and diabetes (Jha et al., 2013). Fibrosis is a common pathological feature of most CKD and ultimately leads to end-stage renal disease, it is characterized by excessive accumulation of extracellular matrix dominated by fibrillar collagen, and myofibroblasts are thought to be the main cell-producing fibrillar collagen. In addition, fibrillar collagen can also be produced by fibroblasts, macrophages, and renal tubular epithelial cells (Panizo et al., 2021; Henderson et al., 2020). Although the etiology of the disease, such as obstruction, can be relieved by surgery, renal fibrosis cannot be eliminated (Henderson et al., 2020; Martínez-Klimova et al., 2019). Therefore, an in-depth and comprehensive investigation of the mechanisms of renal fibrosis to improve the effective treatment of renal fibrosis in obstruction nephropathy remains a priority.

Unilateral ureteral obstruction (UUO) is a well-characterized animal model of renal fibrosis that is thought to mimic the process of human renal fibrosis in an accelerated manner and is used to explore effective biomarkers and novel therapies for progressive renal fibrosis (Chevalier et al., 2009). After a long period of exploration, the pathogenesis of renal fibrosis is clearer. Such mechanisms mainly include (a) infiltration of interstitial macrophages; (b) tubular apoptosis and necrosis; (c) phenotypic transformation of resident renal cells (Panizo et al., 2021; Henderson et al., 2020; Liu, 2006). However, these mechanisms do not fully explain the causes of renal fibrosis, and some questions remain to be addressed: (a) although M2 macrophages are considered to be the most important immune cells in renal fibrosis (Shen et al., 2014; Wang et al., 2018), the mechanisms by which they function are not clear, and additional studies have explained the important role of B cells and T cells in leading to fibrosis (Han et al., 2017; Zhou et al., 2021). A comprehensive analysis of the role of other immune cells in fibrosis is still needed; (b) investigators have used different time nodes in studying the UUO model, including 2, 3, 5, 7, 10, and 14 days. The duration of the obstruction can cause different pathological changes, making timing a tricky issue. The research on the changes in gene expression profiles with obstruction time will provide new ideas for studying fibrosis; (c) the intervention with TGF-β in renal tubular epithelial cells as well as fibrotic cells is used as an *in vitro* model of renal fibrosis (Xiang et al., 2020), however, the differences between *in vivo* and *in vitro* models are not systematically describe; (d) studies on non-coding RNAs in fibrosis still lack comprehensive reports, especially the relationship with immune cells remains to be investigated.

**FIGURE 1:**
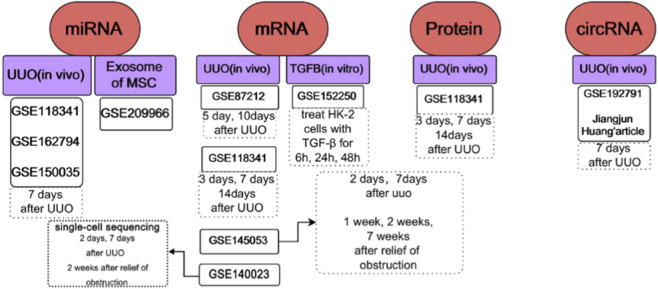
A brief overview of the datasets used in this article.

In recent years, bioinformatics analysis has been widely used for biomarker identification, providing insights into the molecular mechanisms of disease progression. Here, we obtained the proteomics, mRNA sequencing, miRNA sequencing, and circRNA sequencing data from the GEO database on animal models of UUO as well as *in vitro* models (Figure 1). Based on these datasets, we performed a comprehensive analysis of genes and the immune cells with renal fibrosis.

## 2 Methods

### 2.1 Data acquisition and processing

We searched the data on GEO with the keywords “UUO” and “renal fibrosis” and filtered the data with the following criteria: (a) The number of samples in each group is greater than three. (b) Specimens were collected continuously at different obstruction times. We included GSE87212 (Trivedi et al., 2017), GSE118341 (Pavkovic et al., 2019), and GSE145053 (Conway et al., 2020) for studying UUO renal fibrosis at the mRNA level, of which GSE145053 contains sequencing data for 2 days, 7 days after UUO and 1, 2 and 4 weeks after recovery of obstruction. Proteomics data are included for protein level analysis in GSE118341. GSE162794 (Aomatsu et al., 2022), GSE118341, and GSE150035 (Connor et al., 2020) are used for miRNA analysis. Exosomes released by human umbilical cord mesenchymal stem cells (HucMSC-Exo) are reported to have a vital regulatory role in fibrosis (Wang et al., 2019; Zhao et al., 2021). Here, we downloaded the sequencing data of exosomal miRNAs in GSE209966 (Qian et al., 2016) for the study of the role of exosomal miRNAs in fibrosis. We also downloaded data from GSE192791, which includes the sequencing results of circRNAs, and obtained another sequencing data of circRNAs from Jiangjun Huang’s article for verification (Huang J. et al., 2021). In addition, we downloaded data from GSE152250 (Zhang et al., 2020), in which the authors sequenced mRNAs from renal tubular epithelial cells treated with TGF-β for 6, 24, and 48 h, which is a common *in vitro* model of renal fibrosis. Conway et al. (2020) and colleagues’ single-cell sequencing data reported by Conway et al. (2020) were retrieved from the GEO database under accession number GSE140023, and the corresponding analyses were performed using this dataset. We have conducted corresponding analyses using this resource. A detailed description of the data is performed in Figure 1. To ensure the consistency of the analysis, we uniformly downloaded the data in FPKM and TPM forms of mRNA and converted FPKM to TPM for subsequent analysis. We removed low-expressed genes, and the strategy was to retain genes with expression greater than 3 in 10% of the samples. To merge and compare mouse-derived genes with human-derived genes, the Homologene package was used for mouse-human gene homolog conversion. All data processing was based on R software (R 4.0.1).

### 2.2 Differential analyses

We performed differential expression analysis using R software with the Limma package. mRNA sequencing data (TPM) were normalized using the normalizeBetweenArrays function prior to analysis. Differentially expressed mRNAs (DE-mRNAs) were identified with the criteria of |log2 fold change| > 1 and false discovery rate (FDR) < 0.05, with adjusted P values calculated using the Benjamini–Hochberg method to control for multiple testing. For differentially expressed miRNAs (DE-miRNAs) in the UUO fibrosis model, the thresholds were |log2 fold change| > 0.5 and FDR < 0.05, while differentially expressed circRNAs (DE-circRNAs) were screened using |log2 fold change| > 1 and FDR < 0.05. Exosome-derived DE-miRNAs were defined by |log2 fold change| > 1 and FDR < 0.05. Data visualization was performed using the ggplot2 package.

### 2.3 Enrichment analysis

To explore the underlying biological processes of DEGs, the clusterprofile package was used for Kyoto Encyclopedia of Genes and Genomes (KEGG), Gene Ontology (GO) enrichment analysis of DE-mRNAs. Adjusted P values (P. adjust) were calculated using the Benjamini–Hochberg method to control the false discovery rate. Pathways and functional terms with P. adjust < 0.05 were considered statistically significant.

### 2.4 Immuno-infiltration analysis

The Cibersort package was used for immune cell evaluation based on gene sequencing data. The cytogenetic signature gene set was LM22, which contains a total of 547 genes for 22 immune cells (Newm et al., 2019). ANOVA was used to compare differences between groups. P < 0.05 was considered significant.

### 2.5 Fuzzy C-means clustering

The Mfuzz package is a clustering method developed for processing gene expression or protein expression profile data. The core algorithm is based on fuzzy c-means clustering for analyzing temporal trends in gene or protein expression in transcriptomic and proteomic data with time-series characteristics, and for classifying genes or proteins with similar expression patterns to help understand the dynamic patterns of these biological molecules and the linkage to function. Here, we calculated the differential genes for each time point, and the juxtaposition of DE-mRNAs was included in the cluster analysis.

### 2.6 WGCNA

The WGCNA package was used to perform WGCNA analysis based on the genes of 24 samples in GSE145053. The topological overlap matrix was measured based on a pairwise correlation adjacency matrix to estimate the neighborhood similarity between genes. Then the protein co-expression modules of different colors were identified by average linkage hierarchical clustering. We set the power value as 16 according to the method specified by the authors of WGCNA and used the dynamic hybrid tree-cutting algorithm to divide all genes into different modules. We included the relative number of immune cells as phenotypic data and calculated the modules associated with immune cells such as M2, M1, and M0 macrophages. In addition, the correlation between each gene in the module and M2 was verified by combining GSE87212 and GSE118341.

### 2.7 cricRNA/miRNA/mRNA network construction

The circRNA acts as a miRNA sponge and subsequently blocks the function of its corresponding miRNA. We used miRanda to predict the target DE-circRNAs of DE-miRNAs, the multiMiR package for predicting the target mRNA of miRNAs, and we selected experimentally validated genes as the final target. By using Cytoscape, we integrated circRNA-miRNA pairs and miRNA- mRNA pairs to construct a complete circRNA-related regulatory network based on these same miRNA binding sites. According to ceRNA theory, we set the screening conditions to DE-circRNAs with DE-mRNAs co-expression trend and miRNA opposite.

### 2.8 Real-time PCR (RT-PCR)

The *in vitro* and *in vivo* models of renal fibrosis were established by UUO (7 days) and TGF-β intervention in HK-2 cells (48 h). In the *in vivo* model, we used the sham operation group as a control. Refer to our previous article for detailed methodology (Chen et al., 2022). Part of mRNA and miRNA was verified here including Spp1, Havcr1 and several miRNAs by RT-PCR and the method was consistent with our previous study (Tan et al., 2022). GAPDH and U6 were used as internal reference genes for mRNA and miRNA normalization, respectively. Relative expression levels were calculated using the 2^−ΔΔCt method. The primers we used are shown in Table 1.

**TABLE 1:** Primer sequence used in this paper.

Gene	Primer sequences (5′–3′)
miR-30e-5p	CGC​AGT​GTA​AAC​ATC​CTT​GAC (forward)
miR-30a-5p	GCA​GTG​TAA​ACA​TCC​TCG​AC (forward)
miR-30e-3p	GCA​GCT​TTC​AGT​CGG​ATG​T (forward)
let-7g-5p	CGC​AGT​GAG​GTA​GTA​GTT​TG (forward)
miR-455-5p	CAG​TAT​GTG​CCT​TTG​GAC​TAC (forward)
miR-455-3p	GCAGTCCACGGGCAT (forward)
miR-29a-3p	CGCAGTAGCACCATCTGA (forward)
miR-193b-3p	GAACTGGCCCTCAAAGTC (forward)
miR-192a-3p	GCA​GCC​TGT​CAG​TTA​TGT​AG (forward)
Spp1	CTCCATTGACTCGAACGACTC (forward) CAGGTCTGCGAAACTTCTTAGAT (reverse)
Havcr1	TGGCAGATTCTGTAGCTGGTT (forward) AGAGAACATGAGCCTCTATTCCA (reverse)

The reverse primer was obtained from the miRNA RT-PCR, assay Kit.

### 2.9 Immunofluorescence, H&E staining

The tissue specimens were fixed with 4% paraformaldehyde, embedded in paraffin, and cut into 4 μm-thick sections. Sections were dewaxed, heated in citric acid buffer (pH6.0) for 15 min to restore antigen, and then sealed with 5% bovine serum albumin (BSA) for 1 hour. The primary antibody was incubated overnight at 4 °C; The cells were washed with PBS three times and incubated with fluorescent secondary antibody for 1 h. Tissue was stained with Hoechst for 10 min, and the nuclei were observed. Finally, the slices were mounted under a fluorescence microscope and photographed (Olympus Crops, Tokyo, Japan). Primary antibody for immunofluorescence: Havcr1 (1:200, Proteintech), F4/80 (1:200, Proteintech). For H&E staining, the sample slides were first deparaffinized and then hydrated. Subsequently, the samples were treated with a sequence of steps involving hematoxylin, eosin, certain concentrations of alcohol, and xylene to complete the H&E staining.

### 2.10 HucMSC-exo extraction and administration

HucMSCs (catalog: PCS-500-010) are sourced from the Chongqing Stem Cell Biotechnology Research Base. Cells are cultured in DMEM/F12 (Sigma-Aldrich, United States) with 10% fetal bovine serum and 1% penicillin/streptomycin at 37 °C with 5% CO_2_. Upon reaching 80% confluence, the medium is replaced with serum-free, exosome-free medium for 48 h, and the supernatant is collected. The collected supernatant undergoes stepwise centrifugation and filtration, followed by ultracentrifugation to concentrate exosomes. The protein concentration is assessed using the BCA assay, and exosome markers (CD63, Alix) are confirmed by Western blotting. Exosomes are then resuspended in PBS. For electron microscopy, a diluted exosome sample is dropped onto a copper grid, air-dried, fixed, and observed. ZetaView (Particle Metrix GmbH, Germany) tracks exosome quantity and size based on Brownian motion. Diluted exosomes in PBS are measured for size and concentration distribution after 60 s of Brownian motion. A mouse unilateral ureteral obstruction (UUO) model was established. All experimental protocols were approved by the ethics committee of the North Sichuan Medical College (2025047). Eighteen mice were randomly divided into sham, UUO, and UUO + Exo groups. On the first and third days post-surgery, HucMSC-Exo (100 µg) were intravenously injected via the tail vein. On the seventh day post-surgery, kidneys were harvested and placed in formaldehyde solution.

### 2.11 WB

Cells were lysed using RIPA buffer supplemented with protease inhibitors, and total protein concentrations were determined using a BCA protein assay kit. Equal amounts of protein were separated by SDS–PAGE and transferred onto PVDF membranes. After blocking with 5% nonfat milk, membranes were incubated with primary antibodies against E-cadherin and α-SMA overnight at 4 °C, followed by incubation with appropriate HRP-conjugated secondary antibodies. Protein bands were visualized using an enhanced chemiluminescence detection system and quantified by densitometric analysis.

### 2.12 Cell transfection

HK-2 cells were seeded into six-well plates at a density of 5 × 10^5^ cells per well and transfected at approximately 70%–80% confluence using Lipofectamine™ 3000 (Invitrogen, United States) according to the manufacturer’s protocol. miR-30a-5p mimics and corresponding negative controls (NC) were synthesized by Beijing Tsingke Biotech Co., Ltd (The sequences of the mimics and NC are listed in Table 2). Briefly, the oligonucleotides were diluted in Opti-MEM medium and mixed with Lipofectamine 3000 reagent, followed by incubation at room temperature for 20 min to allow complex formation. The transfection complexes were then added to cells in serum-free medium, and after 6 h, the medium was replaced with complete culture medium. Cells were harvested 48 h post-transfection for subsequent analyses.

**TABLE 2:** Sequences of miR-30a-5p mimics and negative control used for transfection.

Gene	Sense (5′-3′)	antisense (5′-3′)
hsa-miRNA-30a-5p-mimcis	UGU​AAA​CAU​CCU​CGA​CUG​GAA​G	UCC​AGU​CGA​GGA​UGU​UUA​CAU​U
Mimcis NC	UUC​UCC​GAA​CGU​GUC​ACG​UAA​A	UUU​ACG​UGA​CAC​GUU​CGG​AGA​A

## 3 Results

### 3.1 Identification of DE-mRNAs in the UUO model

**FIGURE 2:**
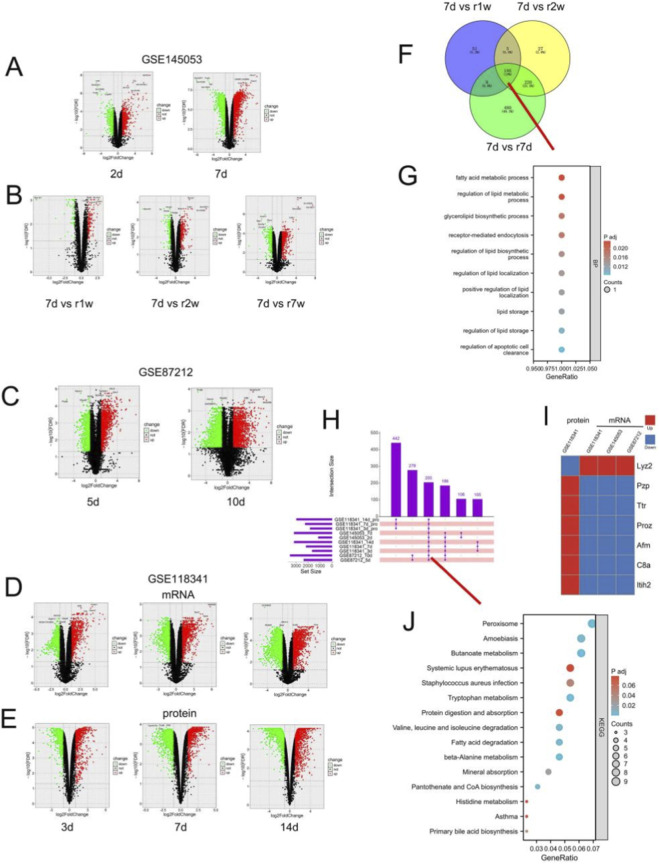
Identification of DE-mRNAs in the UUO kidneys. **(A)** DE-mRNAs in the GSE145053 database (2 days, 7 days after UUO vs. Sham). **(B)** DE-mRNAs after relief of obstruction (1, 2, 7 weeks after relief of obstruction vs. 7 days after UUO). **(C)** DE-mRNAs in the GSE87212 database (5 days, 10 days after UUO vs. Sham). **(D,E)** DE-mRNAs as well as DE-Pros in the GSE118341 database (3 days, 7 days, 14 days after UUO vs. Sham). **(F,G)** The intersection of DE-mRNAs as well as enrichment analysis after the release of obstruction. **(H)** An upset plot showing the intersection of differential genes at different time points in different databases. **(I)** Heatmap showing seven genes with opposite trends at the mRNA vs protein level. **(J)** We specifically performed enrichment analysis of intersection of proteins and mRNAs.

In GSE145053, 1129 and 3143 DE-mRNAs were identified on days 2 and 7 after obstruction, respectively, compared with the sham control group (Figure 2A). In GSE87212, 2340 and 3478 DE-mRNAs were detected on days 5 and 10 (Figure 2C). In GSE118341, 1635, 2149, and 3145 DE-mRNAs were identified at days 3, 7, and 14 after obstruction (Figure 2D). Proteomic data from GSE118341 revealed 1818, 2224, and 2946 differentially expressed proteins (DE-Pros) at the same time points, largely consistent with the RNA-seq results (Figure 2E). GSE145053 also includes data after relief of obstruction (1, 2, and 4 weeks following 7 days of UUO), reflecting renal recovery. Compared with 7-day UUO, 250, 438, and 894 DE-mRNAs were identified at 1, 2, and 4 weeks after relief, respectively (Figure 2B). The number of DE-mRNAs increased with prolonged obstruction. After relief, the expression of some genes gradually returned toward baseline and was mainly enriched in fatty acid metabolism–related pathways (Figures 2F,G). To identify stable biomarkers, we intersected DE-mRNAs across all time points in each dataset during UUO. A total of 391 genes were consistently altered across the three datasets and showed similar expression trends (Figure 2H), suggesting stable involvement in fibrosis progression. Integrating proteomic data with these 391 genes yielded 205 overlapping genes. Among them, seven genes displayed opposite trends at the mRNA and protein levels, while the remaining genes showed consistent expression patterns (Figure 2I), We further explored the functional profiles of the 205 genes. these genes are involved in a variety of metabolism-related pathways, including amino acid metabolism, fatty acid metabolism, etc (Figure 2J).

### 3.2 Differential analysis of mRNA in cellular fibrosis models

**FIGURE 3:**
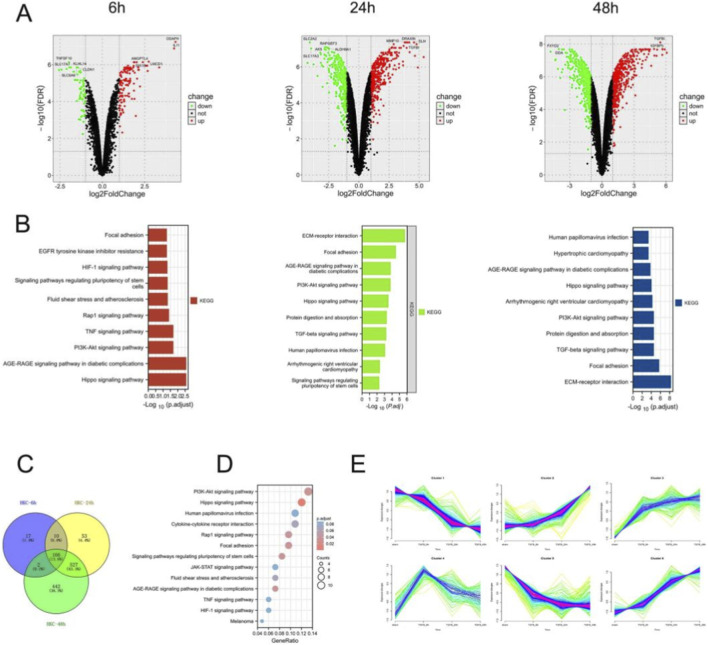
Identification of DE-mRNAs in the *in vitro* fibrosis model. **(A,B)** The DE-mRNAs after the intervention of TGF-βin renal tubular epithelial cells for 6, 24, and 48 h (TGF-β vs. Control). **(B)** Enrichment analysis of DE-mRNAs at 6, 24, and 48 h. **(C,D)** Intersection of DE-mRNAs at different time points and enrichment analysis of 166 intersecting DE-mRNAs. **(E)** C-means clustering revealed the changes of mRNA with the time of TGF-β intervention.

We analyzed the gene expression changes of renal tubular epithelial cells after TGF-β intervention for 6, 24, and 48 h (Figure 3A). Similarly, the number of DE-mRNAs increased with time delay. Comprehensive analysis revealed that the TGF-β signaling pathway showed significant differences at 24 h rather than 6 h and persisted until 48 h (Figure 3B). While 442 genes, including COL6A2, were significantly differentially expressed only at 48 h (Figures 3C,D). Thus we tend to recommend 48 h as the time point for cellular intervention. We took the intersection of the DE-mRNAs at the three time points and found that these genes were mainly associated with the HIPPO signaling pathway, PI3K signaling pathway, and TNF signaling pathway (Figures 3C,D). These DE-mRNAs can be divided into three main categories: 1) the expression level increased with the extension of intervention time; 2) the expression level decreased with time; 3) the expression level turned at 6 h (Figure 3E).

### 3.3 Consistency and differences between *in vitro* and *in vivo* models

**FIGURE 4:**
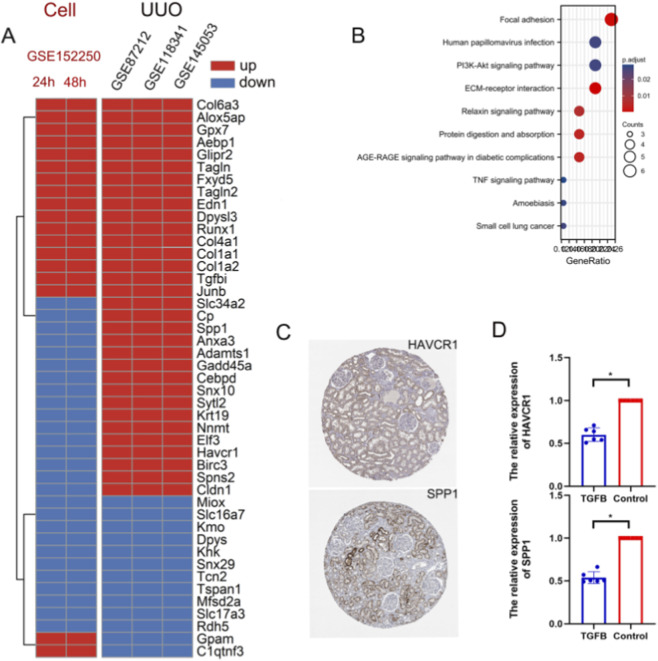
Consistency and differences of *in vitro* and *in vivo* models. **(A)** We took the intersection of DE-mRNAs then we plotted the heat map to show the changes of these genes *in vitro* and *in vivo* models. For example, Havcr1 was upregulated in the UUO kidneys, which was supported by three databases, but significantly downregulated in the *in vitro* fibrosis model. **(B)** Functional enrichment analysis of these intersection genes. **(C)** Expression of Havcr1 and Spp1 in normal kidney. The figures were from the human protein atlas (https://www.proteinatlas.org/). **(D)** RT-PCR confirmed that Havcr1 and Spp1 were indeed significantly decreased in the *in vitro* model (*represents P < 0.05).

TGF-β intervention in renal tubular epithelial cells, as well as fibroblasts, is a commonly used *in vitro* model, but we found that this *in vitro* model can only partially replace *in vivo* experiments. As we found out before, the genes on the TGF-β pathway, PI3K signaling pathway, and fiber-associated pathway showed consistent changes (Figure 4B). However, the *in vitro* model differs from the *in vivo* model in some aspects, which is of concern, implying that the *in vitro* model is to some extent unsuitable as a model of validation (Figure 4A). For example, the Havcr1 was significantly upregulated in the UUO kidneys and was reported to be caused mainly by injured renal tubular epithelial cells (Tang et al., 2021). However, in an *in vitro* model of fibrosis, there was a significant decrease in Havcr1. Spp1 is highly expressed as a secreted protein in UUO kidneys, mainly in tubular epithelial cells as well as macrophages (Huang R. et al., 2021), and is also lowly expressed in the *in vitro* model induced by TGF-β (Figures 4A,C). We performed RT-PCR and verified the trends of mRNA expression such as Havcr1 and Spp1 were consistent with sequencing analysis (Figure 4D), and such contradictory genes included CLDN1, SPNS2, BIRC3, HAVCR1, ELF3, NNMT, KRT19, SYTL2 SNX10, CEBPD, GADD45A, ADAMTS1, ANXA3, SPP1, CP, SLC34A2 (Figure 4A).

### 3.4 Fuzzy clustering analysis reveals the dynamic changes of DE-mRNAs subgroups and their functions

**FIGURE 5:**
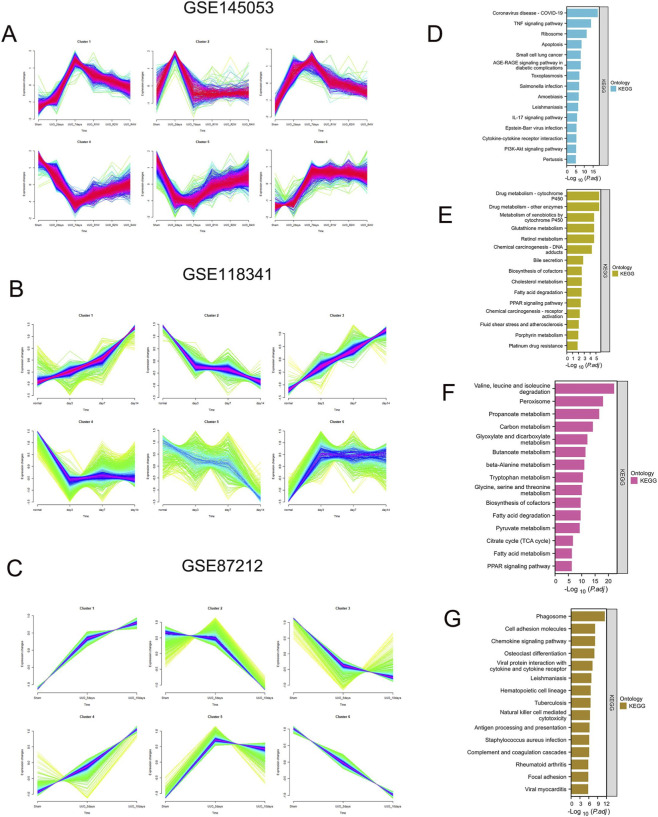
C-means clustering. **(A–C)** Fuzzy c-means clustering identified six distinct temporal patterns of mRNA expression. The x-axis represents the time and status of UUO, while the y-axis represents log2-transformed, normalized intensity ratios in each time point. **(D–G)** enrichment analysis of cluster 1, 3, cluster 2, cluster, 4-5, cluster 6 in the GSE145053. (We incorporated clusters of genes with similar expression trends).

As shown in Figure 5, fuzzy c-means clustering identified six distinct temporal mRNA expression patterns across the GSE145053, GSE118341, and GSE87212 datasets (Figures 5A–C; Supplementary Table 1), indicating dynamic transcriptional changes during UUO. We mainly focused on the trends in GSE145053 because it includes gene expression changes after relief of obstruction. Cluster 1 and Cluster 3 showed similar patterns, characterized by significant upregulation after obstruction followed by gradual decline after relief; therefore, they were merged for subsequent functional enrichment analysis. Cluster 4 and Cluster 5 both exhibited downregulation after obstruction and gradual recovery after relief and were also combined for enrichment analysis. Cluster 2 and Cluster 6 showed relatively independent dynamic expression trajectories. Based on this grouping strategy, KEGG pathway enrichment analysis was performed for the different clusters in GSE145053 (Figures 5D–G). Cluster 1 + 3 was mainly enriched in inflammatory and immune-related pathways, including the TNF signaling pathway, cytokine–cytokine receptor interaction, IL-17 signaling pathway, and infection-related pathways, suggesting that inflammatory activation is a major molecular feature during UUO and can be rapidly alleviated after relief of obstruction (Figure 5D). Cluster 2 was upregulated during the first 2 days after UUO and then rapidly decreased, suggesting an association with acute injury, and was enriched in drug metabolism and fatty acid metabolism pathways (Figure 5E). Cluster 4 + 5 was significantly enriched in fatty acid metabolism, PPAR signaling pathway, peroxisome, and multiple amino acid and energy metabolism pathways. These genes were markedly downregulated after obstruction and increased after relief, suggesting that their function is impaired during renal injury and that they may represent a cluster of protective genes (Figure 5F). Cluster 6 was enriched in cell adhesion molecules, complement and coagulation cascades, and immune regulatory pathways, and this group of genes showed a continuous upward trend (Figure 5G). Overall, different dynamic expression patterns corresponded to different biological functional modules, reflecting the intertwined progression of inflammatory activation and metabolic disorder during UUO.

### 3.5 Multiple immune cells involved in fibrosis

**FIGURE 6:**
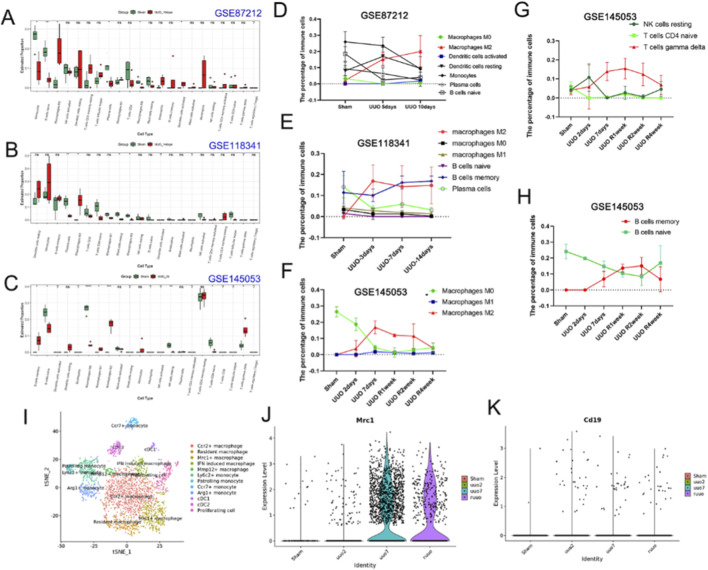
Immune infiltration analysis. **(A–C)** Differences in 22 immune cells in the UUO group compared to the sham group. **(D–H)** Trends in various immune cells with the time of UUO. **(I)** The clusters of myeloid cells were identified by single cell sequencing in UUO. **(J, K)** The number of MRC1 and CD19 positive cells in each group (http://www.ruuo-kidney-gene-atlas.com/). (ns represents P > 0.05, *represents P < 0.05, **represents P < 0.01. ***represents P < 0.001, ****represents P < 0.0001).

We explored the infiltration of immune cells in the UUO model as well as in control kidneys by immune infiltration analysis of the data GSE87212, GSE118341, and GSE145053. The mononuclear macrophage lineage was the immune cell with the highest percentage in the Fibrotic kidney (Figures 6A–C). The three databases were highly consistent in demonstrating that M2 macrophages were significantly hyper-infiltrated in the kidney of UUO (Figures 6A–C) and that M2 macrophage infiltration increased significantly with increasing duration of the obstruction, whereas decreased within 1 week after the obstruction was removed (Figures 6D–F). Relatively insignificant changes were observed in M1 macrophages, and the trend was the opposite in M0 macrophages and M2 macrophages (Figures 6D–F). In addition to macrophages, B cells attracted our attention. The data of GSE145053 showed that B cells belonged to a relatively high proportion of immune cells, and naive B cells steadily decreased with the duration of obstruction and started to recover only in the second week after the release of the obstruction (Figure 6H). In contrast, memory B cells increased in number with prolonged obstruction time and then decreased after relief of the obstruction (Figures 6D,E,H). Other immune cells such as NK cells and T cells may have some differences in the fibrosis process, but the trend is not significant and was less consistent in the three databases (Figure 6G). The results of the single-cell analysis support the findings of the bulk transcriptome sequencing data. MRC1-positive macrophages, resembling M2 macrophages in traditional classification, increased after UUO obstruction and decreased upon relief of the obstruction (Figures 6I,J). Simultaneously, CD19, a marker for B cells, showed a similar trend of elevation after obstruction and reduction upon relief of the obstruction, as indicated by the single-cell sequencing data (Figure 6K).

### 3.6 WGCNA reveals M2 macrophage-related genes

**FIGURE 7:**
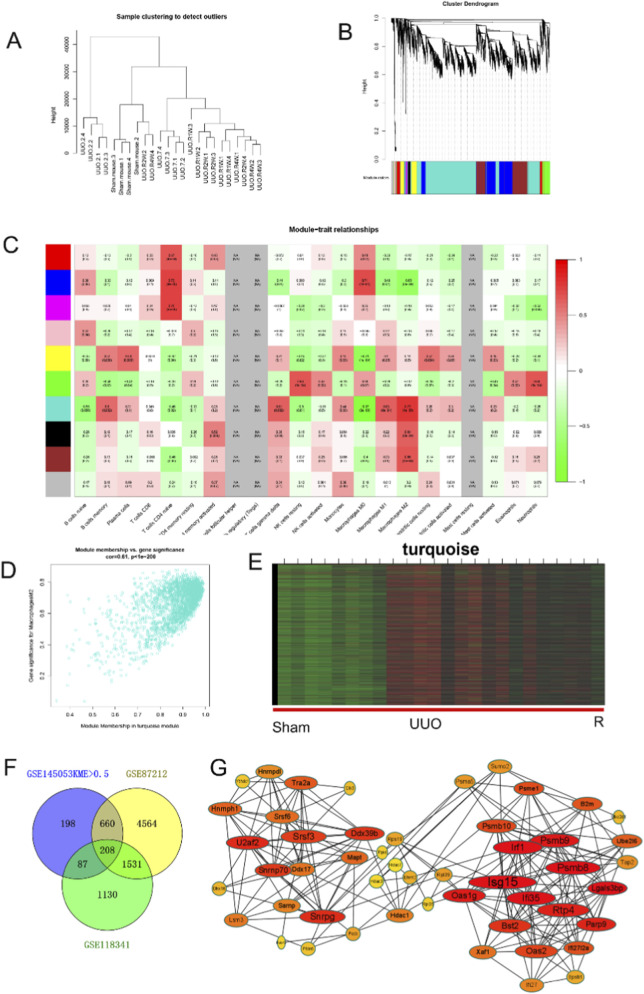
Identification of modules associated with immune cells. **(A)** Clustering dendrogram of samples. **(B)** Dendrogram of genes clustered based on the measurement of dissimilarity (1-TOM). The color band shows the results obtained from the automatic single-block analysis. **(C)** Heatmap of the correlation between the module eigengenes and immune cells. We selected the turquoise block for subsequent analysis. **(D)** Scatter plot of the module eigengenes (MEs) in the turquoise modules. **(E)** Heatmap of genes in the turquoise modules. **(F)** The intersection of the key genes of the turquoise module and the genes associated with M2 macrophages in GSE87212 and GSE118341. **(G)** PPI identified hub genes related to macrophages.

We found above that M2 macrophages play an important role in the fibrosis process. To investigate the possible mechanisms regulating M2 macrophages, the gene modules regulating M2 macrophages were identified by WGCNA. The clustering dendrogram of samples showed successful animal modeling (Figure 7A). We obtained a total of 10 modules after setting the power to 16 and identified that the module with the color turquoise had the highest relevance with macrophages (Figures 7B,C). Then, we screened the key genes in this module with a KME value of 0.9 as the threshold and obtained 1153 genes (the KME value was calculated both for this gene with the module correlation and the correlation with the phenotype) (Figure 7D). The heatmap nicely shows a consistent trend of these genes changing with obstruction time (Figure 7E). In addition, we performed a correlation analysis of all genes in GSE87212 and GSE118341 with M2 macrophage (Supplementary Table 2). By taking the intersection of the key genes of the turquoise module and the genes associated with M2 macrophages in GSE87212 and GSE118341, 208 intersecting genes were obtained, which may play an important role in regulating M2 macrophages (Figure 7F). The string database was used to construct a PPI protein network for these genes. Oas2, Tap2, Bst2, Lgals3bp showed close association as hub genes (Figure 7G), and their functions were also focused on immunomodulation (Li et al., 2022; Lapenna et al., 2017).

### 3.7 Identification of DE-miRNAs as well as cellular origin

**FIGURE 8:**
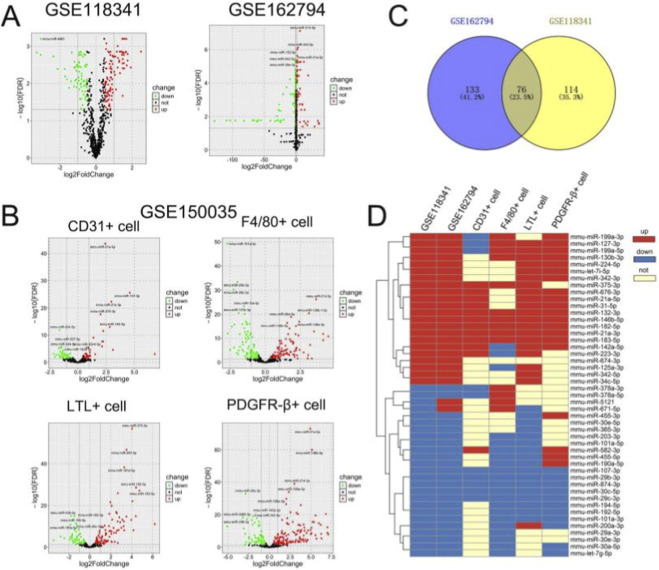
Identification of DE-miRNAs as well as cellular origin. **(A)** Differential analysis of miRNA from GSE118341 as well as GSE162794 (UUO vs. Sham). **(B)** DE-miRNAs in CD31-positive cells (endothelial cells), F4/80-positive cells (macrophages), LTL-positive cells (tubular epithelial cells) during UUO obstruction in GSE209966. **(C)** Intersection of DE-miRNAs in GSE162794 and GSE118341. **(D)** Combined analysis of miRNA in the three databases, heat map displays the cellular origin of DE-miRNAs.

Differential expression analysis of miRNAs between the UUO and sham groups was performed using the GSE118341 and GSE162794 datasets (Figure 8A). The intersection of DE-miRNAs from these two databases was taken, and 76 common DE-miRNAs were obtained (Figure 8C). To explore the specific cellular origin of such DE-miRNAs, we performed differential analysis on the data from GSE152250, which sequenced miRNA by classifying cells into CD31-positive cells (endothelial cells), F4/80-positive cells (macrophages), LTL-positive cells (renal tubular epithelial cells), and PDGFR-β+ cells (fibroblasts). We evaluated 86, 193, 156, and 223 differential miRNAs for CD31-positive, F4/80-positive, LTL-positive, and PDGFR-β+ cells, respectively (Figure 8B). Further, we matched DE-miRNAs of each cell type in GSE152250 with the above 76 DE-miRNAs. 47 miRNAs were matched, and the heatmap suggested that the sequencing results from different databases were consistent, with overall elevated levels of miRNAs often contributed by elevated miRNAs of one or several cellular origins (Figure 8D). Five miRNAs (miRNA-132-3p, miRNA-146b-5p, miRNA-182-5p miRNA-21a-3p, miRNA-183-5p) were upregulation at the overall level and were elevated inside all four cell types. miRNA-125a-3p, miRNA-342-5p, and miRNA-34c-5p were upregulation only in renal tubular cells. miRNA-199a-3p was elevated in both macrophages as well as fibroblasts (Figure 8D).

### 3.8 HucMSC-exo regulate UUO fibrosis progression via miRNA

**FIGURE 9:**
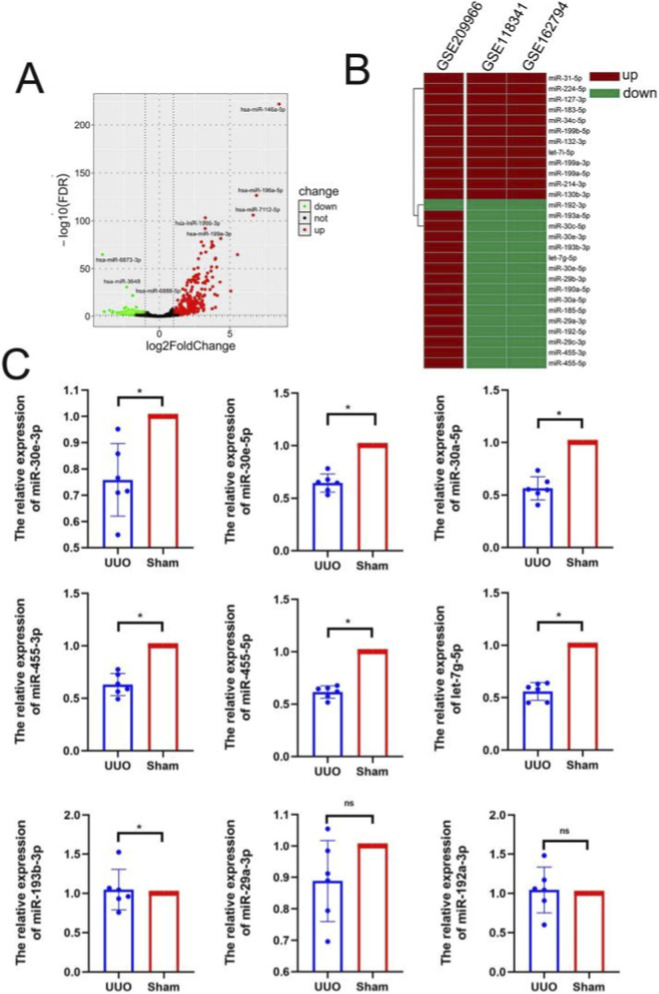
The miRNAs derived from HucMSC-Exo that may have therapeutic effects on fibrosis. **(A)** DE-miRNAs in HucMSC-Exo (MSC vs. MRC-5). **(B)** Combination of DE-miRNAs in HucMSC-Exo as well as in the UUO kidneys. **(C)** RT-PCR validation of miRNAs (UUO vs. Sham). (Data are presented as mean ± SD. Statistical significance was determined using one-way ANOVA followed by appropriate *post hoc* tests. ns represents P > 0.05, *represents P < 0.05).

HucMSC-Exo were reported to have a reparative effect on fibrosis by miRNA, and here we explored the role of miRNAs in exosomes. We downloaded sequencing data from GSE209966 and found that 268 of these miRNAs were significantly upregulated and 243 were downregulated in HucMSC-Exo (Figure 9A). Combined analysis with DE-miRNAs in the UUO kidneys revealed that 15 miRNAs were downregulated in UUO kidneys but significantly upregulated in HucMSC-Exo (Figure 9B), implying that these miRNAs may have an inhibitory effect on fibrosis. Meanwhile, we conducted RT-PCR to validate, which showed that miRNA-30, miRNA-455, and miRNA-let-7g-5p decreased significantly after obstruction (Figure 9C).

### 3.9 Differential analysis of circRNA

**FIGURE 10:**
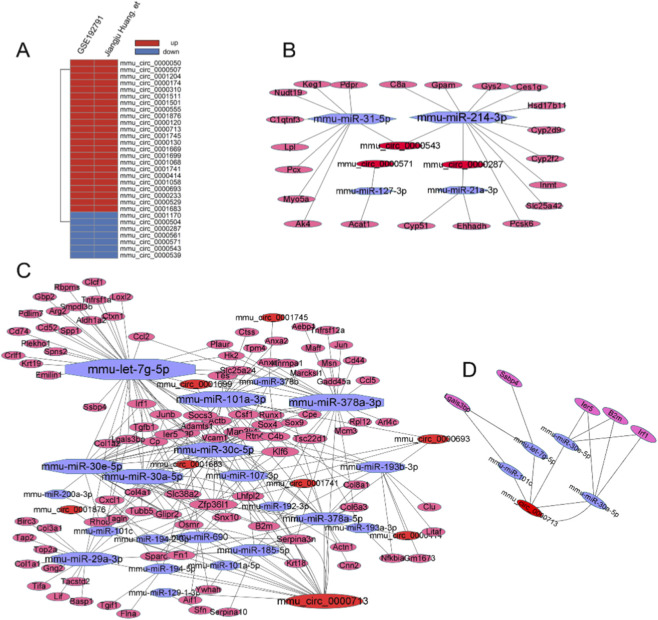
CeRNA network. **(A)** Identification of DE-circRNAs in the two independent datasets (UUO vs. Sham). **(B)** Downregulated circRNA associated ceRNA network. **(C)** Upregulated ceRNA associated ceRNA network. **(D)** Macrophage associated ceRNA network.

We analyzed the sequencing data from GSE192791 and circRNA sequencing data from Jiangju Huang. 77 and 103 DE-circRNAs were identified, and a total of 30 common DE-circRNAs remained after taking the intersection of the two data (Figure 10A). These 30 DE-circRNAs showed the same variation in both databases. Among them, there are 23 circRNAs upregulated and seven circRNAs downregulated (Figure 10A). The four circRNAs with a fold change greater than two were mmu_circ_0001501, mmu_circ_0000529, mmu_circ_0000120, and mmu_circ_0000414 (Figure 10A).

### 3.10 Upstream regulatory mechanisms of fibrosis

We conducted an integrated analysis of DE-mRNAs, DE-miRNAs, and DE-circRNAs during UUO-induced renal fibrosis. Since circRNAs can function as miRNA sponges and miRNAs negatively regulate target mRNAs, we constructed two regulatory networks: an upregulated circRNA–downregulated miRNA–upregulated mRNA network, and a downregulated circRNA–upregulated miRNA–downregulated mRNA network. We found that upregulated circRNAs were predominant in the UUO fibrosis model, and correspondingly, miRNAs were predominantly downregulated (Figures 10B,C). Three downregulated circRNAs, circ-0000287, circ-0000571, circ-0000543 targeted miRNA-214-3p, miRNA-31-5p, miRNA-1273p, miRNA-21a-3p to regulate LPL, C8a. As shown in the figure (Figure 10B), the upregulated circRNAs were widely involved in the regulation of fibrosis, and the most important circRNAs include circRNA_0000713, circRNA_0001699, etc. CircRNA-0000713 target multiple miRNAs including miRNA-let-7g-5p, miRNA-30c-5p, miRNA-30a-5p, miRNA-378a-3p. Meanwhile, miRNA-let-7g-5p, miRNA-378a-3p, miRNA-30c-5p, and other miRNAs were involved in regulating TGF-β and col8a1 as the most widely connected miRNAs (Figure 10C).

### 3.11 The ceRNA mechanism is involved in macrophage regulation

We have previously demonstrated the genes associated with M2 macrophages and explored the specific role of the ceRNA network in immune regulation. We took the target genes of miRNA in the ceRNA network to intersect with macrophage-related genes. ceRNA mechanism mediated the expression of five macrophage-related genes, namely, B2m, Irf1, ler5, Lgals3bp, and Ssbp4. circ_0000713 regulated the expression of multiple macrophage-related mRNAs through multiple pathways (Figure 10D). The networks of Circ_0000713-miRNA-let7g-5p/miRNA-30-Lgals3bp/Ssbp4 is of particular interest to us. miRNA-let-7g-5p, miRNA-30e-5p, and miRNA-30a-5p are not only downregulated in UUO, regulated by circRNA, targeting multiple DE-mRNAs, but interestingly, it is significantly upregulated in HucMSC-Exo.

### 3.12 Exosomes attenuate renal fibrosis by modulating macrophage infiltration and fibrotic markers

**FIGURE 11:**
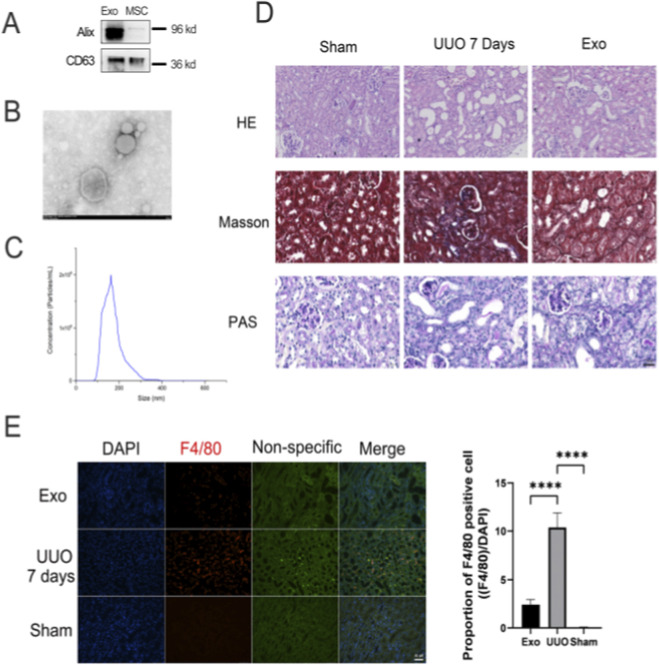
HucMSC-Exo regulate macrophage infiltration. **(A)** Western blot (WB) was performed to identify the expression of exosome markers. **(B)** Transmission electron microscopy (TEM) was utilized to observe the morphology of HucMSC-Exo (100 K magnification). **(C)** Representative results of NTA of HucMSC-Exo. **(D)** H&E staining, PAS staining and Masson’s trichrome staining. **(E)** Immunofluorescence was performed to detect the expression of the macrophage marker F4/80. To visualize the location of macrophages in the kidney, non-specific staining was applied to show renal tissues. (Data are presented as mean ± SD. Statistical significance was determined using one-way ANOVA followed by appropriate *post hoc* tests. ****represents P < 0.0001).

Although exosome-based therapies for renal fibrosis have been extensively explored, their regulatory effects on macrophages remain underexamined. In this study, we preliminarily investigated the impact of mesenchymal stem cell-derived exosomes (MSC-Exos) on renal pathology and macrophage dynamics in a murine unilateral ureteral obstruction (UUO) model. Exosomes were successfully isolated and characterized from MSCs (Figures 11A–C).

**FIGURE 12:**
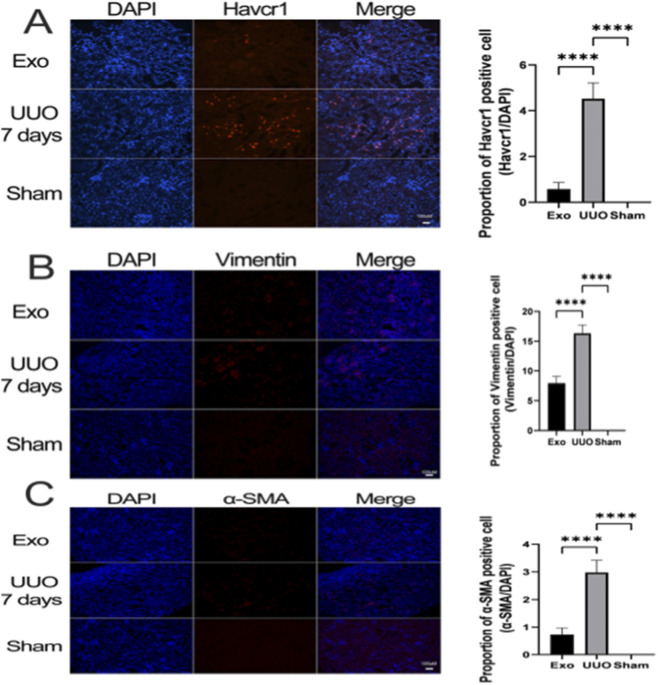
HucMSC-Exo regulate renal tubular epithelial cell injury. **(A)** Immunofluorescence was performed to detect the expression of Havcr1. **(B)** Immunofluorescence was performed to detect the expression of Vimentin. **(C)** Immunofluorescence was performed to detect the expression of a-SMA. (Data are presented as mean ± SD. Statistical significance was determined using one-way ANOVA followed by appropriate *post hoc* tests. ****represents P < 0.0001).

Histopathological analysis via H&E and PAS staining revealed intact renal architecture in the Sham group, whereas the UUO group exhibited tubular dilation, epithelial injury, and interstitial collagen deposition. Notably, exosome intervention significantly mitigated these pathological alterations (Figure 11D). Masson’s trichrome staining further confirmed that collagen fiber accumulation, prominent in the interstitial regions of UUO kidneys, was markedly reduced in exosome-treated mice (Figure 11D). macrophage infiltration, predominantly localized in the interstitium, was significantly elevated post-UUO but markedly attenuated by exosome treatment (Figure 11E). Immunofluorescence staining of fibrosis-related markers—Havcr1, vimentin, and α-SMA—demonstrated robust expression in UUO kidneys, aligning with bioinformatics predictions. Strikingly, exosome administration suppressed the upregulation of these markers (Figures 12A–C).

### 3.13 MiR-30a-5p attenuates TGF-β–induced epithelial–mesenchymal transition

**FIGURE 13:**
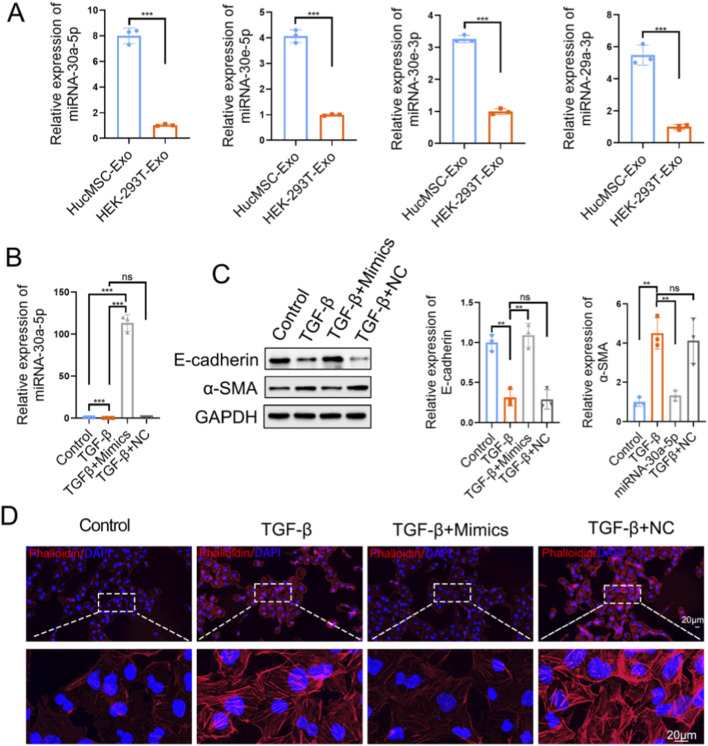
miR-30a-5p attenuates TGF-β–induced epithelial–mesenchymal transition. **(A)** Quantitative PCR analysis showing the relative expression levels of selected miRNAs (miR-30a-5p, miR-30e-5p, miR-30e-3p, and miR-29a-3p) in exosomes derived from mesenchymal stem cells (MSCs) and HEK-293T cells. **(B)** Relative expression of miR-30a-5p in cells under different treatment conditions. **(C)** Western blot analysis and quantitative densitometry of epithelial marker E-cadherin and mesenchymal marker α-SMA under the indicated conditions. **(D)** Representative immunofluorescence images showing cytoskeletal organization (red) and nuclei (blue) under the indicated treatments. (Data are presented as mean ± SD from at least three independent experiments. Statistical significance was determined using one-way ANOVA followed by appropriate *post hoc* tests. ns represents P > 0.05; **represents P < 0.01; ***represents P < 0.001).

We isolated exosomes and performed RT–PCR analysis. Compared with exosomes derived from HEK-293T cells, mesenchymal stem cell (MSC) derived exosomes contained significantly higher levels of some miRNAs, including miR-30a-5p, miR-30e-5p, miR-30e-3p, and miR-29a-3p (Figure 13A). Given the marked enrichment of miR-30a-5p, it was selected for further investigation. Cells were transfected with miR-30a-5p mimics or the corresponding negative control (NC) using a lipid-based transfection reagent. Quantitative RT-PCR confirmed successful transfection, with miR-30a-5p expression increased by approximately 100 fold (Figure 13B). Functionally, TGF-β treatment induced epithelial–mesenchymal transition (EMT), as indicated by reduced E-cadherin expression and increased α-SMA levels, whereas overexpression of miR-30a-5p partially reversed these changes (Figure 13C). Consistent with these findings, immunofluorescence analysis showed that TGF-β stimulation promoted cytoskeletal remodeling and stress fiber formation, which were markedly attenuated by miR-30a-5p overexpression (Figure 13D). Collectively, these results suggest that miR-30a-5p enriched in MSC-derived exosomes exerts a protective effect against TGF-β–induced EMT and cytoskeletal reorganization.

## 4 Discussion

UUO models are widely used to study the pathogenesis and diagnostic markers of renal fibrosis (Chevalier et al., 2009). With the advent of second-generation sequencing, the study of fibrosis has been facilitated by the comprehensive characterization of gene expression through multiple omics analysis. Here, we perform a comprehensive analysis of 12 sequencing data from the GEO dataset and published articles at the mRNA, protein, miRNA, and circRNA levels for cells as well as animal models (UUO) of renal fibrosis.

Integrated transcriptomic and proteomic analyses identified 205 overlapping fibrosis-associated genes enriched mainly in metabolic pathways, highlighting metabolic dysregulation as a key feature of renal fibrosis (Zhao et al., 2020). It is noteworthy that there are five genes with opposite trends at the mRNA level to the protein level. This may be a false positive due to the lack of multiple database validation of the protein level data, but it is also possible that the difference is due to post-transcriptional modifications, as we found multiple m6a methylated genes to be differentially expressed, consistent with previous reports (Xu et al., 2021; Xing et al., 2022). The time of UUO was identified as an important influencing factor of gene expression by our study. Through cluster analysis, we found that the UUO model was unstable in the early stages (intervention for 2–3 days), with metabolic disturbances being the main event in this phase. It revealed that 7 days was an earlier time to reach a steady state. The clustering analysis mainly classified genes into four categories. These four different gene expression trends are important for studying the mechanisms of fibrosis. To better understand the changing trend of each gene, we put the clustering results into the Supplementary Table 1.

In contrast to UUO as an *in vivo* model to study renal fibrosis, TGF-β intervention in tubular epithelial cells as well as fibroblasts is often used as an *in vitro* model to study fibrosis (Kang et al., 2015). Our analysis suggests that the difference at 48 h is the most pronounced, with multiple fibrosis indicators as well as pathways showing significant differences only at 48 h. Note that although both the *in vitro* model and the *in vivo* model showed consistent changes in genes on the TGF-β pathway and PI3K signaling pathway, *in vitro* models of fibrosis are not a complete alternative to *in vivo* models. This is because the *in vitro* model differs from the *in vivo* model in some aspects, for example, Havcr1 was significantly upregulated in the UUO model and was reported to be caused mainly by injured renal tubular epithelial cells (Tang et al., 2021). Nevertheless, in the *in vitro* model of fibrosis (tubular epithelial cells), the renal injury molecule was significantly decreased. Similarly, Spp1 was highly expressed in UUO and was low in the TGF-β-stimulated *in vitro* model (Huang R. et al., 2021). The increase of Spp1 and Havcr1 in the UUO model has been supported by abundant studies, while its decrease in the *in vitro* model has not been reported. Therefore, we verified by RT-PCR *in vitro.* It was proved that the *in vitro* model did lead to the decrease of Spp1 and Havcr1. These shreds of evidence highly warn us that the *in vitro* model should be used cautiously in some cases. As for why this contradiction occurs, we suspect that there are two explanations. One is the complexity of the signaling pathway. The current *in vitro* model only mainly considers the TGF-β pathway, while *in vivo* it is much more complicated. Perhaps the activation of other pathways will lead to the increase of those genes. The other one is the interaction between cells. For example, macrophages are likely to transfer certain genes to epithelial cells via the exosome (Huang R. et al., 2021). Anyway, this is an interesting line of research.

M2 macrophages are the most studied cells in renal fibrosis, and we found a significant increase of M2 macrophages in the UUO kidneys, accounting for approximately 20% of all immune cells. To investigate the regulatory mechanism of M2 macrophages, we performed WGCNA, correlation analysis, and PPI. We identified Oas2, Tap2, Bst2, and Lgals3bp as hub genes with strong relevance to M2 macrophages. Oas2 is associated with interferon and macrophages (Gaurav et al., 2021) Tap2 affects the survival of macrophages (Lapenna et al., 2017). Most notably, Lgals3bp, also known as M2bp, is a secreted glycoprotein found in the extracellular matrix of various tissues (Kyrousi et al., 2021; Capone et al., 2020). It has been reported to serve as a serum diagnostic marker for a variety of fibrosis and to have an effect on the fibrotic process (Shirabe et al., 2018). It has been shown that Lgals3bp secreted by OS cells binds to Lgals3 ligands on M1 macrophages and induces the secretion of Hspa11 through Akt phosphorylation, inhibiting the killing activity of macrophages (Li et al., 2022). Therefore, it is of interest whether Lgals3bp influences the process of fibrosis through M2 macrophage during renal fibrosis. In addition to macrophages, naive B cells are among the predominant immune cells. A study showed that the accumulation of early B cells accelerated the mobilization and infiltration of monocytes/macrophages, which exacerbated the fibrosis caused by acute renal nephropathy (Han et al., 2017). It is evident that B cells play an important role in fibrosis, while most studies have focused on macrophages and less on B cells, which is worthy of further study.

miRNA repression of mRNA expression has been extensively studied as an upstream regulatory mechanism of mRNAs. Based on miRNA sequencing of two independent data, we identified 76 miRNAs that changed consistently in both studies and found miRNAs that changed relatively consistently over time in the UUO model. Connor et al. first proposed the classification of CD31-positive cells (endothelial cells), F4/80-positive cells (macrophages), LTL-positive cells (tubular epithelial cells), and PDGFR-β+ cells (fibroblasts), and examined miRNA expression in these cells in the UUO kidneys (Connor et al., 2020). Combined analysis of these three independent sequencing data, we identified 47 DE-miRNAs. We plotted heat maps that showed which cells contributed to the elevation of each miRNA. Renal tubular epithelial cells did contribute the largest proportion of miRNA upregulation, for example, miRNA-21-5p was highly expressed in tubular epithelial cells and fibroblasts, while one study showed that exosome- derived miR-21 from tubular cells activated fibroblasts by targeting PTEN (Zhao et al., 2021). Similarly, in the UUO kidneys, miRNA-199a-5p was highly expressed in tubular epithelial cells, whereas a study suggested that 199a-5p promoted the transformation of tubular epithelium towards a fibrotic phenotype (Yang et al., 2017). It is important to note that some miRNAs are highly expressed only in one type of cell, which requires a careful selection of cell lines for *in vitro* experiments. We have repeatedly stressed that in many cases the choice of TGF-β intervention on renal tubular epithelial cells as an *in vitro* model of renal fibrosis must be made with caution. In addition to the reasons mentioned above for mRNA expression contradiction, some miRNAs were not elevated in renal tubular epithelial cells, such as miRNA-199a-3p. Our identification of overall DE-miRNAs as well as cell-specific DE-miRNAs provides an opportunity to study cell-to-cell interactions.

Considering the importance of miRNA in regulating fibrosis and miRNA is an abundant substance in exosomes. We believe that exosome-derived miRNAs could play a role in repairing fibrosis. HucMSC-Exo have been shown to repair fibrosis (Qiu et al., 2022). Combined with sequencing data from HucMSC-Exo, we identified miRNAs that may affect fibrosis. In particular, we focused on these miRNAs that were downregulated in the UUO kidneys but were abundant in HucMSC-Exo for the reason that they may alleviate effects on fibrosis. These miRNAs include miRNA-29, miRNA-30, miRNA-455 families, etc. These findings are in line with previous studies, one of which found that exosome-mediated miR-29 transfer reduced muscle atrophy and renal fibrosis in mice (Wang et al., 2019). Another study demonstrated that HucMSC-Exo alleviate systemic sclerosis, a multiorgan fibrotic disease, through miR-29a-3p (Rozier et al., 2021). In this study, we preliminarily explored the potential role of miR-30a-5p in alleviating renal fibrosis.

miRNA plays a vital role in fibrosis. circRNA, as an important regulatory substance of miRNA, also has similar biological activities. circRNA_37492 can act as a potential ceRNA to alleviate obstructive renal fibrosis (Cheng et al., 2022), and circRNA_33702 promotes renal fibrosis by targeting the miR-29b-3p/WISP1 pathway (Ai et al., 2022). Overall, there are relatively few studies on circRNAs in renal fibrosis, here we integrated two independent data to identify DE-circRNAs. Twenty-three circRNAs were upregulated and seven circRNAs were downregulated and further, we constructed the ceRNA network. Circ_0000713-miRNA-let7g-5p/miRNA-30-Lgals3bp/Ssbp4 axis is noteworthy. circRNA circ_0000713 targets miRNA-let-7g-5p, miRNA-30e-5p, miRNA- 30a-5p. And all these 3 miRNAs can target macrophage-related genes, such as lgals3bp, Ssbp5, Ier5. Here we must emphasize again the importance of miRNA-let-7g-5p, miRNA-30e-5p and miRNA- 30a-5p, which was not only downregulated in UUO kidneys, regulated by circRNA, targeting multiple macrophage-related differentially expressed mRNAs, interestingly, it was significantly upregulated in HucMSC-Exo.

In conclusion, we synthetically analyzed multi-omics data from 12 databases in this study to provide a comprehensive analysis of renal fibrosis. Our main contributions performed as follows: (a) differences in mRNA, as well as temporal trends, were identified; (b) we clarified the similarities and differences between *in vivo* and *in vitro* models; (c) we performed a comprehensive analysis of immune cells, identifying M2-related genes and other fibrosis-related immune cells; (d) we constructed circRNA-miRNA-mRNA networks and identified their relationship with immune cells; (e) we identified the DE-miRNAs and sort out the cellular origin of miRNAs, providing new insights into the role of miRNAs and intercellular interactions; (f) we identified miRNAs in the HucMSC-Exo that may have a mitigating effect on fibrosis, providing insights into HucMSC-Exo for the treatment of fibrosis. Although some parts have not been verified by experiments, it should be noted that we integrated multiple independent data for analysis, thus we believe that our analysis results are of reference significance.

## Data Availability

The original contributions presented in the study are included in the article/Supplementary Material, further inquiries can be directed to the corresponding author.
